# Immunohistochemistry for CCR4 C‐terminus predicts *CCR4* mutations and mogamulizumab efficacy in adult T‐cell leukemia/lymphoma

**DOI:** 10.1002/cjp2.180

**Published:** 2020-10-06

**Authors:** Keiichiro Fujii, Yuma Sakamoto, Ayako Masaki, Takayuki Murase, Yukie Tashiro, Kentaro Yonekura, Atae Utsunomiya, Asahi Ito, Shigeru Kusumoto, Shinsuke Iida, Ryuzo Ueda, Takashi Ishida, Hiroshi Inagaki

**Affiliations:** ^1^ Department of Pathology and Molecular Diagnostics, Graduate School of Medical Sciences Nagoya City University Nagoya Japan; ^2^ Department of Pathology Imamura General Hospital Kagoshima Japan; ^3^ Department of Dermatology Imamura General Hospital Kagoshima Japan; ^4^ Department of Hematology Imamura General Hospital Kagoshima Japan; ^5^ Department of Hematology and Oncology, Graduate School of Medical Sciences Nagoya City University Nagoya Japan; ^6^ Department of Tumor Immunology Aichi Medical University School of Medicine Nagakute Japan; ^7^ Department of Immunology Nagoya University Graduate School of Medicine Nagoya Japan

**Keywords:** ATL, CCR4, immunohistochemistry, mogamulizumab, prognosis

## Abstract

Mogamulizumab targets extracellular N‐terminal domain of CCR4, which is expressed in most adult T‐cell leukemia/lymphoma (ATL) cases. Recently, we reported that *CCR4* C‐terminal gain‐of‐function mutations were frequent in ATL cases, and a subgroup with these mutations who were treated without allogenic hematopoietic stem cell transplantation (HSCT) and with mogamulizumab‐containing [HSCT (−) and mogamulizumab (+)] regimens had a superior survival rate. Although these mutations are most likely a biomarker for predicting a strong response to mogamulizumab, their detection is time‐consuming and costly. A more convenient screening tool may be necessary in the clinical setting. In this study, the clinicopathological importance of immunohistochemistry for the CCR4 N‐terminus (CCR4‐N‐IHC) and C‐terminus (CCR4‐C‐IHC) was examined in a large ATL cohort (*n* = 92). We found that CCR4‐C‐IHC, but not CCR4‐N‐IHC, was inversely correlated with the *CCR4* mutation status. In ATL patients negative for CCR4‐C‐IHC, a subgroup treated with HSCT (−) and mogamulizumab (+) regimens showed a significantly better prognosis. In addition, CCR4‐C‐IHC was found to be a useful marker for high‐sensitivity screening of the *CCR4* mutational status (87%). The present study suggests that CCR4‐C‐IHC may be useful for identifying ATL patients harboring mutated *CCR4* who may benefit from the superior efficacy of mogamulizumab‐containing regimens and that CCR4‐C‐IHC may be a rapid and cost‐efficient tool for screening for *CCR4* mutation status.

## Introduction

Adult T‐cell leukemia/lymphoma (ATL) is a T‐cell neoplasm caused by human T‐cell lymphotropic virus type 1 (HTLV‐1) [1, 2, 3]. Patients, especially those with an aggressive variant (acute, lymphoma, or unfavorable chronic subtype), have a very poor prognosis. C‐C chemokine receptor 4 (CCR4) is expressed by tumor cells of most ATL patients. Mogamulizumab, an anti‐CCR4 monoclonal antibody, which recognizes the extracellular N‐terminus of CCR4, has offered therapeutic benefit to ATL patients [4, 5, 6]. Recently, we reported that in ATL patients with *CCR4* mutations, a subgroup treated without allogenic hematopoietic stem cell transplantation (HSCT) and with mogamulizumab‐containing [HSCT (−) and mogamulizumab (+)] regimens had significantly better survival rates [7]. These *CCR4* mutations are usually of the nonsense or frameshift type, are mainly found in the area corresponding to the C‐terminus of the CCR4 protein, and may truncate the amino acid sequences in this terminus [7, 8, 9]. The C‐terminus is considered to be a target site of arrestin, which plays an important role in CCR4 internalization [10]. Thus, loss of the CCR4 C‐terminus may inhibit CCR4 internalization, leading to long lasting expression of the CCR4 extracellular N‐terminus, a target site for mogamulizumab [8, 9].

*CCR4* mutations are most likely a biomarker for predicting whether mogamulizumab treatment will be of high efficacy [7]. However, detection of the mutations is time‐consuming and costly. Alternative methods for detecting these mutations may be necessary in the clinical setting. Immunohistochemistry, which is both rapid to perform and cost‐effective, is a useful alternative method. In this study employing a large number of ATL cases, we performed immunohistochemistry for the CCR4 N‐terminus (CCR4‐N‐IHC) and CCR4 C‐terminus (CCR4‐C‐IHC) and analyzed the clinicopathological significance of CCR4 expression of the respective termini. In addition, we examined whether CCR4‐IHC was useful as a rapid and low‐cost test to screen ATL cases for their *CCR4* mutation status.

## Materials and methods

### ATL patients

We analyzed tumor samples from 92 patients with ATL. Among the ATL patients enrolled in our previous study [7], the patients whose affected ATL tissues were available for immunohistochemical analysis were enrolled in the present study. Thus, *CCR4* mutation status was already analyzed in all of the patients as previously described. Diagnosis and assignment of clinical ATL subtypes were conducted according to Japan Lymphoma Study Group recommendations [11]. ATL tumor samples were obtained from Nagoya City University Graduate School of Medical Sciences (Nagoya, Japan) and Imamura General Hospital (Kagoshima, Japan). In all 92 ATL cases, formalin‐fixed, paraffin‐embedded (FFPE) samples (lymph nodes, 37; skin, 35; bone marrow, 9; and others, 11) were prepared and clinical data were obtained. The ATL patients enrolled in this study were treated in various different ways, as determined at each investigator's clinical discretion. A modified LSG15‐like protocol, or a combination of cyclophosphamide–doxorubicin–vincristine–prednisolone (CHOP‐like regimen) with or without mogamulizumab, was initially administered to many patients with acute or lymphoma subtypes [12, 13]. It was planned that relatively younger patients (≤70 years) would receive subsequent allogeneic HSCT while in remission, because it is considered unlikely that ATL patients with acute or lymphoma subtypes will survive long term without this procedure [14, 15, 16, 17]. Mogamulizumab use within 2–3 months before allogeneic HSCT was strictly prohibited in order to avoid increased HSCT‐related mortality [18]. Patients with chronic or smoldering subtypes were mostly managed on a careful watch‐and‐wait basis until disease progression to acute or lymphoma subtypes. Some patients received lenalidomide [19]. The overall response rate (ORR) was assessed in the patients treated with HSCT (−) and mogamulizumab (+) regimens according to the response criteria described elsewhere [20]. This study was approved by the Ethical Board of Nagoya City University and Imamura General Hospital and conducted in accordance with the Declaration of Helsinki.

### Immunohistochemistry for N‐ and C‐termini of CCR4

We performed immunohistochemistry on FFPE tumor sections from ATL cases and employed two different antibodies against CCR4. One was a monoclonal mouse antibody (KM‐2160, POTELIGEO® TEST IHC, Kyowa Medical Cooperation, Tokyo, Japan), which recognizes an extracellular epitope (amino acids 12–29) located at the N‐terminus of CCR4. The other was a polyclonal rabbit antibody (Novus Biologicals, Littleton, CO, USA), which recognizes an intracellular epitope (amino acids 335–360) located at the C‐terminus of CCR4. Immunohistochemical signals were evaluated semi‐quantitatively by the signal intensity, using a scoring scale of 0, 1+, 2+, and 3+ [21]. Areas where the immunohistochemical signals were most intense were evaluated. Scoring of CCR4‐IHC was performed by two expert hematopathologists (AM and HI) and, when scoring was discordant, a consensus score was reached. The specificity of the antibody for the N‐terminus of CCR4 and that for the C‐terminus were confirmed by absorption test. Sections were incubated using each antibody preabsorbed with synthetic peptide (Eurofins genomics, Tokyo, Japan) corresponding to the epitope of the antibody at a concentration of 1 nmol/ml diluted antibody.

### Statistical analysis

Differences between the two groups were compared using the Chi‐cross test, Fisher's exact test, or Mann–Whitney *U*‐test. Survival was evaluated by the Kaplan–Meier method and compared using the log‐rank test. The start date for assessing progression‐free survival (PFS) and overall survival (OS) was the day on which the tumor sample was obtained. PFS was defined as the time to progression, relapse, or death resulting from any cause, whichever occurred first. For survival of patients treated with HSCT, the start date was the day on which HSCT was performed. For the survival of patients treated with mogamulizumab‐containing regimens, the start date was the day on which the first dose of antibody was administered [7]. All analyses were carried out with JMP version 14.2.0 (SAS, Cary, NC, USA), and *p* < 0.05 (two‐tailed) was considered statistically significant.

## Results

### Prognostic significance of *CCR4* mutations in the present ATL cohort

Using direct sequencing and/or the SNaPshot multiplex assay, *CCR4* mutations in the region corresponding to the CCR4 C‐terminus were analyzed in this study cohort. *CCR4* mutations were detected in 31/92 patients (33.7%): C329* (*n* = 8), Q330* (*n* = 4), Y331* (*n* = 7), Q336* (*n* = 5), R323fs (*n* = 2), F326fs (*n* = 1), C329fs (*n* = 1), and S345fs (*n* = 3). There was no significant difference between patients with or without *CCR4* mutations with regard to age, sex, clinical variant, Eastern Cooperative Oncology Group performance status, the serum soluble interleukin‐2 receptor level, serum‐adjusted calcium, serum albumin, Ann Arbor stage, white blood cell counts, hemoglobin, or platelet counts. There was also no significant difference in treatment strategies between patients with or without *CCR4* mutations. Therefore, no obvious clinical features distinguished ATL patients with *CCR4* mutations from those without. The present ATL cohort showed that the survival rate was significantly impacted by *CCR4* mutations in the patients treated with HSCT (−) and mogamulizumab (+) regimens (all patients including patients with an aggressive variant). In this cohort, no significant difference was found in survival between patients treated with mogamulizumab monotherapy (*n* = 21) and those treated by mogamulizumab plus other chemotherapy (*n* = 15) (*p* = 0.689, log‐rank test). The association between *CCR4* mutations and a superior survival rate was not found in any other patient group. The ORRs, assessed in the ATL patients treated with HSCT (−) and mogamulizumab (+) regimens, were 89% and 67% in ATL patients positive and negative for *CCR4* mutations, respectively (see supplementary material, Table S1).

### Immunohistochemistry for the CCR4 N‐terminus and CCR4 C‐terminus and correlation with *CCR4* gene mutations

**Figure 1:**
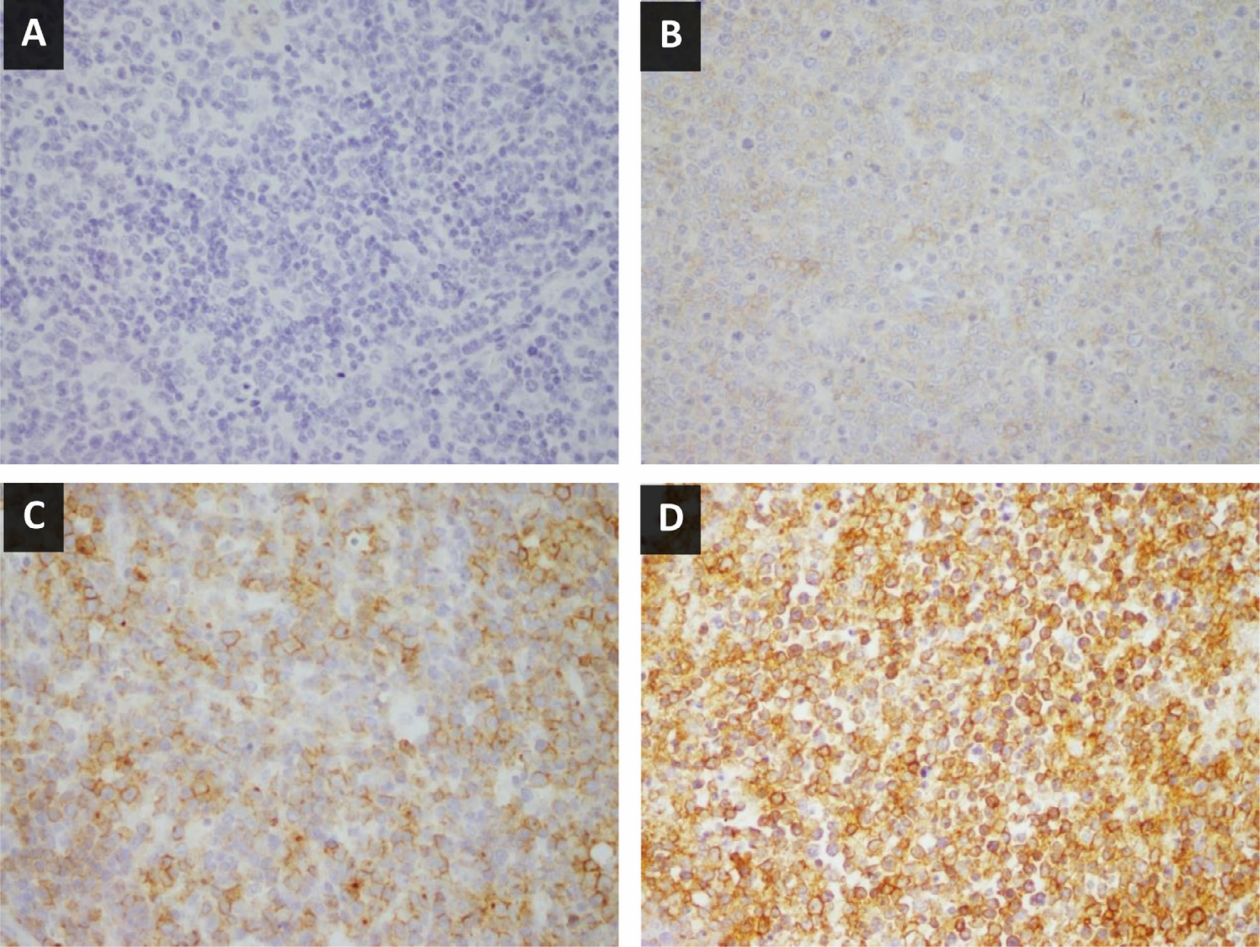
Representative CCR4 C‐terminus expression in ATL cases with intensities scored 0 (negative, A), 1+ (weak, B), 2+ (moderate, C), and 3+ (strong, D).

Immunohistochemistry using antibodies for the N‐ and C‐termini of CCR4 (CCR4‐N‐IHC and CCR4‐C‐IHC, respectively) was performed in 92 ATL cases. Immunostaining for the N‐ and C‐termini of CCR4 was completely abolished by preabsorption of the antibodies with synthetic peptides corresponding to the respective epitopes. ATL cases scored as 0, 1+, 2+, or 3+ for CCR4‐N‐IHC numbered 2, 7, 25, and 58, respectively (Table 1 and see supplementary material, Figure S1). On employing any of the cutoff points for CCR4‐N‐IHC, no significant correlation was obtained between the CCR4‐N‐IHC scores and the presence of *CCR4* mutations. ATL cases scored as 0, 1+, 2+, and 3+ for CCR4‐C‐IHC numbered 4, 26, 22, and 40, respectively (Table 2). Representative images of CCR4‐C‐IHC are shown in Figure 1. When the cutoff point was set between 0/1+ and 2/3+ for CCR4‐C‐IHC, CCR4‐C‐IHC showed an inverse association with the presence of *CCR4* mutations (*p* < 0.0001), and the sensitivity and specificity were calculated to be 68% and 85%, respectively. This cutoff point was considered to be useful for confirmation of the presence of *CCR4* mutations. When the cutoff point was set between 0/1/2+ and 3+ for CCR4‐C‐IHC, CCR4‐C‐IHC showed an inverse association with the presence of *CCR4* mutations (*p* < 0.0001), and the sensitivity and specificity were calculated to be 87% and 59%, respectively. With a high sensitivity of 87%, this cutoff point was considered to be useful for screening for the presence of *CCR4* mutations.

**Table 1:** *CCR4* mutations and immunohistochemistry for the CCR4 N‐terminus.

	*CCR4* mutations	
CCR4‐N‐IHC	Absent (*n* = 61)	Present (*n* = 31)
0	1	1
1+	5	2
2+	17	8
3+	38	20

**Table 2:** *CCR4* mutations and immunohistochemistry for CCR4 C‐terminus.

	*CCR4* mutations	
CCR4‐C‐IHC	Absent (*n* = 61)	Present (*n* = 31)
0	2	2
1+	7	19
2+	16	6
3+	36	4

Thirteen cases showed discordant results between CCR4‐C‐IHC and *CCR4* mutations: in CCR4‐C‐IHC, four cases with *CCR4* mutations were scored high (3+) and nine cases with no *CCR4* mutation were scored low (0/1+). These 13 discordant cases showed no particular clinicopathological features, and when we explored whole *CCR4* exon sequences for mutations using the Sanger method, no mutation was found.

#### Prognostic significance of immunohistochemistry for the N‐terminus and C‐terminus of CCR4

**Figure 2:**
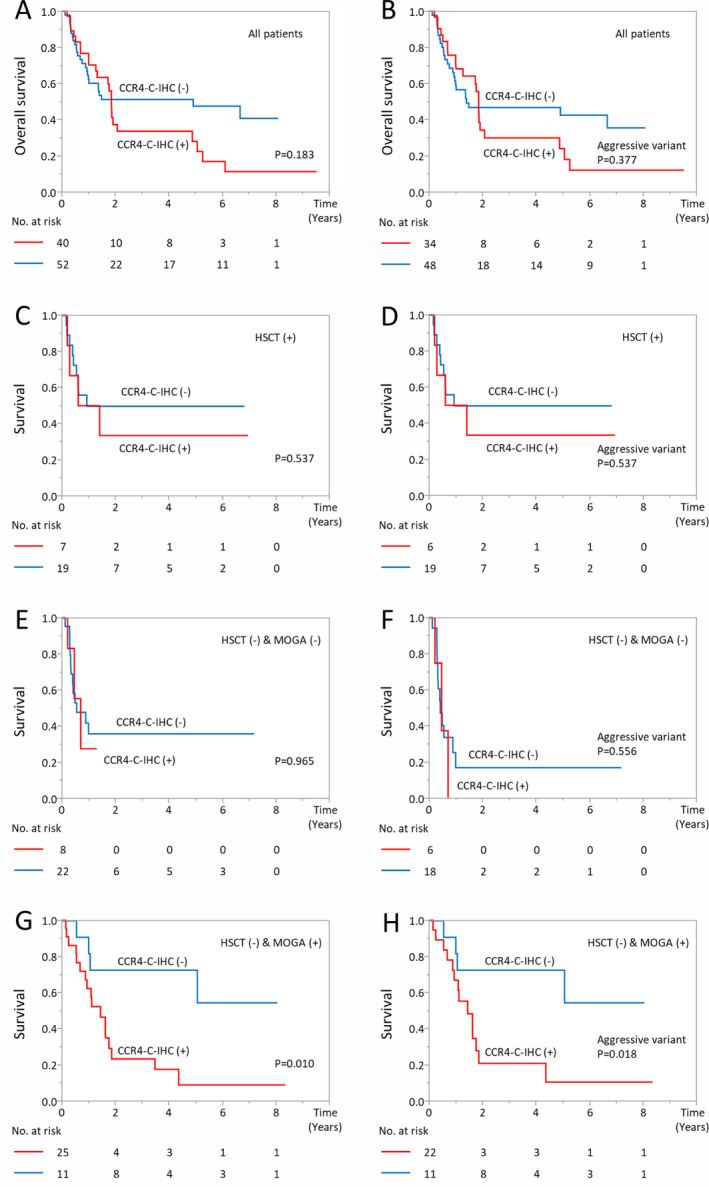
Survival of patients with ATL stratified according to CCR4‐C‐IHC findings (negative, 0/1/2+ versus positive, 3+). (A) The 5‐year OS of the 40 patients positive for CCR4‐C‐IHC and 52 patients negative for CCR4‐C‐IHC was 28.0% (95% CI, 13.9–48.3%) and 47.6% (95% CI, 33.2–62.4%), respectively. (B) Among the patients with ATL with an aggressive variant (*n* = 82), the 5‐year OS of those positive for CCR4‐C‐IHC (*n* = 34) or negative for CCR4‐C‐IHC (*n* = 48) was 24.0% (95% CI, 10.5–45.8%) and 42.5% (95% CI, 27.9–58.5%), respectively. (C) Among the 26 patients with ATL who received allogeneic HSCT, the 5‐year survival from the day of allogeneic HSCT in the seven patients positive for CCR4‐C‐IHC and 19 negative for CCR4‐C‐IHC was 33.3% (95% CI, 8.4–73.2%) and 49.4% (95% CI, 27.7–71.3%), respectively. (D) Among the 25 patients with ATL with an aggressive variant who received allogeneic HSCT, the 5‐year survival from the day of allogeneic HSCT in the six patients positive for CCR4‐C‐IHC and 19 negative for CCR4‐C‐IHC was 33.3% (95% CI, 8.4–73.2%) and 49.4% (95% CI, 27.7–71.3%), respectively. (E) Among the 30 patients with ATL who did not receive any MOGA‐containing treatment or allogeneic HSCT, the 5‐year OS in the eight positive for CCR4‐C‐IHC and 22 negative for CCR4‐C‐IHC was not reached and 35.7% (95% CI, 17.5–59.3%), respectively. (F) Among the 24 patients with ATL with an aggressive variant who did not receive any MOGA‐containing treatment or allogeneic HSCT, the 5‐year OS in the six positive for CCR4‐C‐IHC and 18 negative for CCR4‐C‐IHC was not reached and 16.8% (95% CI, 4.5–46.4%), respectively. (G) Among the 36 patients with ATL who received MOGA‐containing treatment but no allogeneic HSCT, the 5‐year survival from the day of the first dose of antibody in the 25 patients positive for CCR4‐C‐IHC and 11 negative for CCR4‐C‐IHC was 8.7% (95% CI, 1.4–38.4%) and 72.7% (95% CI, 41.4–91.0%), respectively. This difference was statistically significant. (H) Among the 33 patients with ATL with an aggressive variant who received MOGA‐containing treatment but no allogeneic HSCT, the 5‐year survival from the day of the first dose of antibody in the 22 patients positive for CCR4‐C‐IHC and 11 negative for CCR4‐C‐IHC was 10.3% (95% CI, 1.7–43.5%) and 72.7% (95% CI, 41.4–91.0%), respectively. This difference was also statistically significant. Survival curves were compared using the log‐rank test, and the *P* value is indicated in each panel. MOGA, mogamulizumab.

The prognostic significance of CCR4‐N‐IHC and CCR4‐C‐IHC was analyzed in ATL patients stratified by treatment (HSCT and mogamulizumab‐containing regimen). CCR4‐N‐IHC had no prognostic impact with any of the cutoff points employed (data not shown). On the other hand, CCR4‐C‐IHC (cutoff point between 0/1/2+ and 3+, Table 2) had a prognostic impact on a subgroup treated with HSCT (−) and mogamulizumab (+) regimens (Figure 2). When both indolent and aggressive ATL patients were included, patients scored as 0/1/2+ for CCR4‐C‐IHC showed survival rates superior to those scored as 3+ for CCR4‐C‐IHC (*p* = 0.010, log‐rank test) with the 5‐year survival being 72.7% and 8.7%, respectively. The ORRs assessed in this cohort were 100% and 60% in ATL cases scored as 0/1/2+ and 3+ for CCR4‐C‐IHC, respectively (see supplementary material, Table S1). When ATL patients with an aggressive variant were included in the analysis, the difference in survival rates was more extreme with the 5‐year survival being 72.7% and 10.3%, respectively (*p* = 0.018, log‐rank test). Patient characteristics for CCR4‐C‐IHC showed an association of the score of 0/1/2+ with only female gender (Table 3). Treatment strategies were not apparently different between patients scored CCR4‐C‐IHC 0/1/2+ and those scored 3+ (see supplementary material, Table S2). When the other cutoff point was employed, CCR4‐C‐IHC failed to show a prognostic impact on a subgroup treated with treated with HSCT (−) and mogamulizumab (+) regimens.

**Table 3:** Patient characteristics and CCR4‐C‐IHC.

		CCR4‐C‐IHC		*P* value
Factor		0/1/2+ (*n* = 52)	3+ (*n* = 40)	
Age	≤70	40	31	1.000
	>70	12	9	
Sex	Female	33	15	0.020
	Male	19	25	
Clinical variant	Indolent	4	6	0.322
	Aggressive	48	34	
ECOG PS	0 and 1	39	28	0.641
	2, 3 and 4	13	12	
sIL‐2R (U/mL)	≤20,000	32	24	1.000
	>20,000	17	14	
Serum Ca (mg/dL)	≤11	43	32	0.550
	>11	6	7	
Serum Alb (g/dL)	≥3.5	31	29	0.358
	<3.5	18	10	
Stage	I and II	7	1	0.132
	III and IV	45	39	
WBC (/μL)	Mean	11,338	21,941	0.114
	Median	7560	9650	
	Range	900–60,600	2500–232,100	
Hb (g/dL)	Mean	12.3	13.0	0.118
	Median	12.5	13.3	
	Range	7.9–15.7	8.9–16.3	
Plt (10^3^/μL)	Mean	220	198	0.275
	Median	209	194	
	Range	29–602	28–443	

PS, performance status.

## Discussion

Recently, we showed that the survival rate was significantly superior in an ATL subgroup with *CCR4* gene mutations who were treated with HSCT (−) and mogamulizumab (+) regimens [7]. In this study, an immunohistochemical analysis was performed using two different antibodies (one against the CCR4 N‐terminus and the other against the C‐terminus). CCR4‐C‐IHC (cutoff point, 0/1/2+ versus 3+) was useful in identifying ATL patients who had responded well to mogamulizumab‐containing therapies. CCR4‐C‐IHC was also useful in estimating *CCR4* mutation status with a high sensitivity of 87% (cutoff point, 0/1/2+ versus 3+) and a high specificity of 85% (cutoff point, 0/1+ versus 2/3+).

The most interesting finding of this study was that CCR4‐C‐IHC was inversely associated with the presence of *CCR4* mutations. Since all *CCR4* mutations in the present study were of the nonsense or frameshift type and were localized in the area corresponding to the C‐terminus of the CCR4 protein, decreased CCR4‐C‐IHC expression was most probably explained by amino acid truncation in the C‐terminus of this protein in the tumor cells. Similar to the superior survival of ATL patients with *CCR4* mutations, we found that a lower expression on CCR4‐C‐IHC was associated with a better prognosis in ATL patients when mogamulizumab‐containing regimens were administered. However, there were some cases with discordance between CCR4‐C‐IHC and *CCR4* mutations. In these cases, we examined whole *CCR4* gene sequences and found no additional mutations in any case. This discordance is difficult to explain but the most plausible explanation may involve variable CCR4 expression by a nonmutated allele of the *CCR4* gene. As *CCR4* mutations are usually found on one *CCR4* allele [21], the other allele was considered to remain intact and functional. In addition, the CCR4 expression level may be influenced by various other factors including concentrations of the CCR4 ligands, TARC and MDC [22, 23], which may be present in the microenvironment around ATL cells. In a previous study using flow cytometry, surface CCR4 expression was mildly increased in tumor cells with mutated *CCR4* genes, compared with tumor cells with wild type *CCR4* genes [9]. However, no significant association was found between CCR4‐N‐IHC and *CCR4* mutations in this study. We speculate that the discrepancy may be explained by sensitivity of immunohistochemical analysis, which is usually less than that of flow cytometry [24, 25].

The clinicopathological significance of CCR4‐C‐IHC may vary depending on the reason for use and the cutoff points employed. When the cutoff point was set between 0/1+ and 2/3+, CCR4‐C‐IHC may be useful as a marker for estimating *CCR4* mutation with a high specificity (85%). On the other hand, when the cutoff point was set between 0/1/2+ and 3+, CCR4‐C‐IHC may be useful as a marker for predicting prognosis in ATL patients treated with HSCT (−) and mogamulizumab (+) regimens and can also be used to screen for *CCR4* mutations with a high sensitivity (87%), followed by accurate molecular testing. Although a sensitivity of 87% is not extremely high, we believe that it is useful for screening purposes in the clinical setting. Trastuzumab is a powerful molecularly targeted drug and, for HER2‐positive breast cancer, adjuvant trastuzumab with chemotherapy is a standard treatment [26]. Immunohistochemistry for HER2 is widely employed for screening the *HER2* gene status, often before molecular testing [27]. The sensitivity of immunohistochemistry for HER2 in previous studies was reported to be 87.5–95.6% [28, 29], which seems to be comparable to the degree of sensitivity of our CCR4‐C‐IHC for ATL (87%).

Although this study offers significant observations regarding CCR4‐C‐IHC for patients with ATL receiving mogamulizumab‐containing treatment, several limitations must be noted. The present study had no eligibility criteria, which resulted in enrolment of heterogeneous ATL patients who received various different treatments based on each investigator's clinical discretion. Of the patients who received mogamulizumab‐containing treatment, some were on mogamulizumab monotherapy, others had different combination therapies. All these variables could have affected the present results.

In conclusion, we showed that CCR4‐C‐IHC was inversely correlated with *CCR4* mutations in ATL patients and was useful in identifying ATL patients who may benefit from the superior efficacy of mogamulizumab‐containing regimes. We also showed that CCR4‐C‐IHC was a useful marker for estimating mutational status in the *CCR4* gene. Although nearly 100 ATL cases were analyzed in this study, the number of *CCR4* mutation‐positive cases decreased to 31 and that of patients treated with HSCT (−) and mogamulizumab (+) regimens decreased to 36. Large‐scale studies stratified to therapeutic regimens are warranted to clarify the clinicopathological significance of CCR4‐C‐IHC for estimating *CCR4* mutations and for predicting the clinical response to mogamulizumab‐containing therapy.

## Author contributions statement

KF, YS and HI designed the research. KF, YS, AM, TM, YT, KY, AI and SK performed the experiments. KF, YS, AU, SI, RU, TI and HI analyzed and interpreted data. All authors wrote and approved the manuscript.

## Supporting information


**Figure S1.** Representative immunohistochemical CCR4 N‐terminus expression in ATL cases
**Table S1.** Overall response rates for *CCR4* mutations and CCR4‐C‐IHC in ATL patients treated with HSCT (−) and mogamulizumab (+) regimens
**Table S2.** CCR4‐C‐IHC and mogamulizumab‐containing treatment
