# Supplementary material for: Immunohistochemistry for CCR4 C‐terminus predicts CCR4 mutations and mogamulizumab efficacy in adult T‐cell leukemia/lymphoma
Source: J Pathol Clin Res. 2020 Oct 6;7(1):52–60. doi: 10.1002/cjp2.180 (PMC7737778; doi:10.1002/cjp2.180)
Supplement: Supplementary file 1 — Figure S1. Representative immunohistochemical CCR4 N‐terminus expression in ATL cases Table S1. Overall response rates for CCR4 mutations and CCR4‐C‐IHC in ATL patients treated with HSCT (−) and mogamulizumab (+) regimens Table S2. CCR4‐C‐IHC and mogamulizumab‐containing treatment [file CJP2-7-52-s001.docx]

**Immunohistochemistry for CCR4 C-terminus predicts *CCR4* mutations and mogamulizumab efficacy in adult T-cell leukemia/lymphoma**

Fujii K *et al*, *J Pathol Clin Res*, DOI 10.1002/cjp2.180

**Supplementary Material**


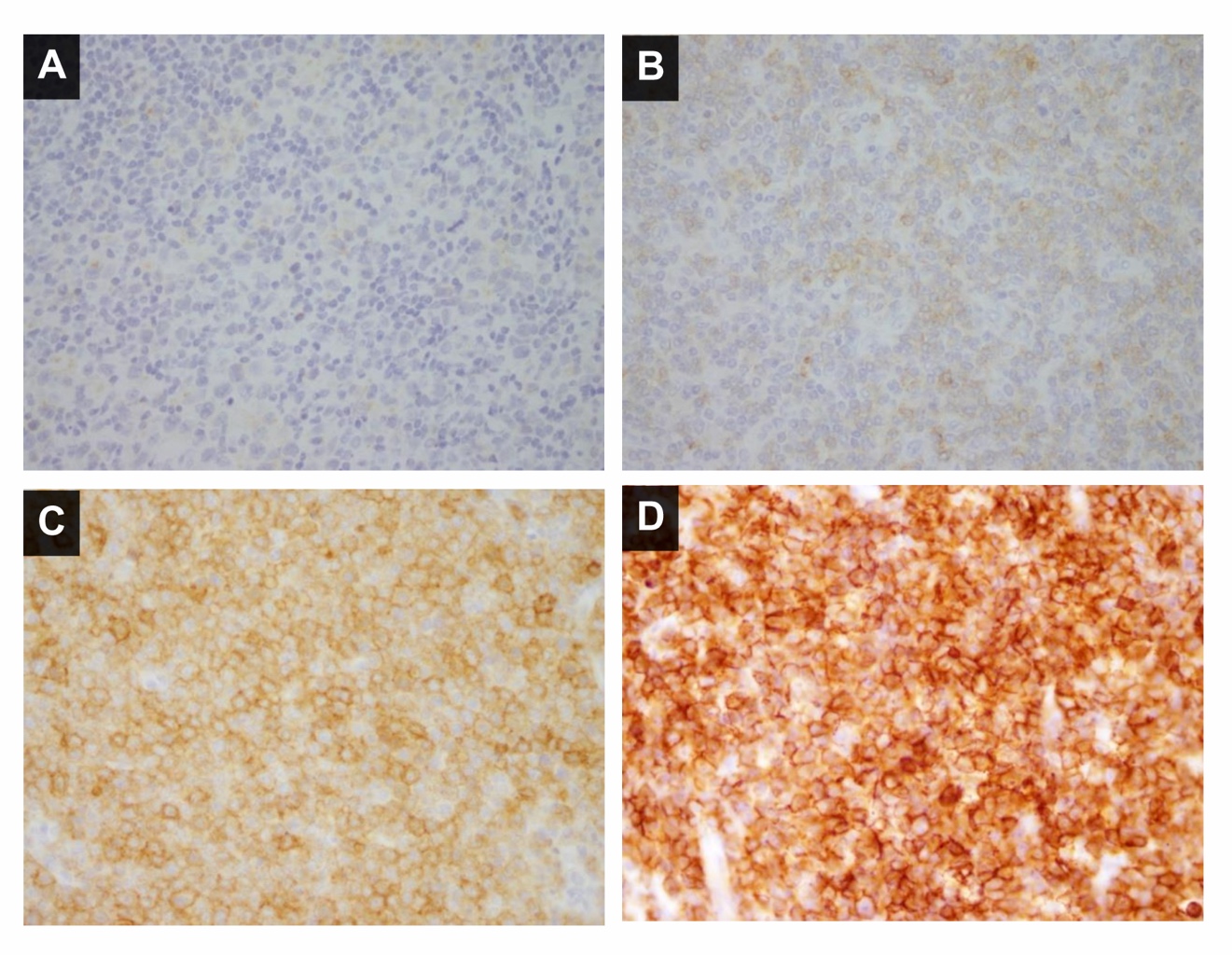


**Figure S1.** Representative immunohistochemical CCR4 N-terminus expression in ATL cases with intensities scored 0 (negative, A), 1+ (weak, B), 2+ (moderate, C), and 3+ (strong, D).

**Table S1.** Overall response rates (ORRs) for *CCR4* mutations and CCR4-C-IHC in ATL patients treated with HSCT (-) and mogamulizumab (+) regimens

| Cohort | Total (n) | CR (n) | PR (n) | SD (n) | PD (n) | ORR |
| --- | --- | --- | --- | --- | --- | --- |
| *CCR4* mutations (+) | 9 | 8 | 0 | 0 | 1 | 8/9 (89%) |
| *CCR4* mutations (-) | 27 | 11 | 7 | 5 | 4 | 18/27 (67%) |
| CCR4-C-IHC (0/1/2+) | 11 | 10 | 1 | 0 | 0 | 11/11 (100％) |
| CCR4-C-IHC (3+) | 25 | 9 | 6 | 5 | 5 | 15/25 (60％) |

IHC, immunohistochemistry; ATL, adult T-cell leukemia/lymphoma; HSCT, hematopoietic stem cell transplantation; CR, complete response; PR, partial response; SD, stable disease; and PD, progressive disease.

**Table S2.** CCR4-C-IHC and mogamulizumab-containing treatment

|  | CCR4-C-IHC | |  |
| --- | --- | --- | --- |
| Treatment strategies | 0/1/2+ (n=11) | 3+ (n=25) | P |
| MOGA monotherapy | 7 | 14 | 0.651 |
| MOGA + CHOP like regimen | 0 | 3 |  |
| MOGA + mLSG15 like regimen | 3 | 5 |  |
| MOGA + other regimens | 1 | 3 |  |

MOGA, mogamulizumab; CHOP, cyclophosphamide, doxorubicin, vincristine, and prednisolone; mLSG15, modified mLSG15, VCAP (vincristine, cyclophosphamide, doxorubicin, and prednisolone), AMP, and VECP (vindesine, etoposide, carboplatin, and prednisolone)

IHC, immunohistochemistry
